# cGMP at the centre of attention: emerging strategies for activating the cardioprotective PKG pathway

**DOI:** 10.1007/s00395-018-0679-9

**Published:** 2018-05-15

**Authors:** Min Park, Peter Sandner, Thomas Krieg

**Affiliations:** 1 Experimental Medicine and Immunotherapeutics, Department of Medicine, University of Cambridge, Addenbrookes Box 110, Cambridge, CB2 0QQ UK; 2 Bayer AG, Drug Discovery, Wuppertal, Germany

**Keywords:** Ischaemia/reperfusion, Cardioprotection, cGMP

## Abstract

The nitric oxide (NO)–protein kinase G (PKG) pathway has been known for some time to be an important target for cardioprotection against ischaemia/reperfusion injury and heart failure. While many approaches for reducing infarct size in patients have failed in the past, the advent of novel drugs that modulate cGMP and its downstream targets shows very promising results in recent preclinical and clinical studies. Here, we review main aspects of the NO–PKG pathway in light of recent drug development and summarise potential cardioprotective strategies in which cGMP is the main player.

## Introduction

Since the initial discovery that nitric oxide (NO) is the endothelial-derived relaxing factor, the last few decades have seen intensive research directed toward understanding this signal molecule and its intracellular signalling cascades. Numerous preclinical studies have shown promising results suggesting a cardioprotective role of NO signalling and revealed the NO–cGMP–PKG cascade as the responsible signalling pathway [6, 27, 28, 30, 39, 63]. Indeed numerous studies have demonstrated that disruption of this pathway leads to various pathological changes in the heart, including vascular and ventricular dysfunction, fibrosis and hypertrophy. Although not all studies are in accordance with this optimistic view on the NO–cGMP–PKG system, targeting this pathway has gained much attention, particularly from scientists keen to develop an efficient drug to treat heart failure. In this brief review, we will focus on pharmacological aspects of the cGMP–PKG pathway with updates from recent preclinical and clinical studies in relation to its direct effect on the heart. We also refer readers to excellent recent reviews providing in-depth overview of NO–cGMP–PKG pathway such as [40].

## Cardioprotective cGMP–PKG pathway

NO and natriuretic peptides (NPs) are the two known classes of upstream molecules that can trigger the cGMP–PKG pathway. NO initiates the signalling pathway by activating soluble guanylate cyclase (sGC), a heterodimeric enzyme consisting of α- and β-subunits with a prosthetic heme moiety, which catalyses cGMP synthesis. On the other hand, NPs, such as ANP (atrial NP), BNP (brain NP) and CNP (C-type NP), activate particulate GC (pGC) present in the plasma membrane to produce cGMP. Both sGC and pGC generate the same second messenger cGMP, but the downstream effects of cGMP can be strikingly different depending on its subcellular localization. The elevated intracellular level of cGMP exerts its physiological actions largely by targeting cGMP-dependent protein kinase (PKG). In mammals PKG-I is the primary kinase responsible for transducing the physiological effects in the cardiovascular system. PKG-Iα and PKG-1β have different N-termini derived from alternative splicing. It is reported that PKG-Iα is ten times more sensitive to cGMP than PKG-1β. Also, due to a unique cysteine residue (Cys 42) in PKG-Iα, it can be activated by thiol-oxidation independently of cGMP [4]. This cGMP-independent form of PKG-1α activation is able to lower blood pressure through vasodilation, but its effect in cardiomyocytes is unknown.

A number of studies have suggested that there are multiple downstream effectors of cGMP-PKG-I in the cardiovascular system. For example, an elevated level of cytosolic Ca^2+^ can result in increased cardiomyocyte inotropy but it is also known to be a lethal cause of reperfusion injury to cardiomyocytes. Several targets have been proposed as downstream PKG effectors regulating Ca^2+^ homeostasis in cardiomyocytes [11]. One of the proposed mechanisms is that PKG-I phosphorylates phospholamban at Ser16, which subsequently activates SR Ca^2+^-ATPase (SERCA). Activated SERCA increases re-uptake of Ca^2+^ into the sarcoplasmic reticulum (SR) and attenuates the cytosolic Ca^2+^ during systole [31]. Also, it was demonstrated that cGMP-mediated PKG-I activation induces opening of mitoK_ATP_ channels residing on the inner membrane of mitochondria, and the subsequent increased K^+^ influx causes alkalinisation of the matrix which increases H_2_O_2_ production from complex I. The increased H_2_O_2_ activates PKC-ε and consequently protects cardiomyocytes from cell death by inhibiting the opening of mitochondrial permeability transition pores (MPTP) [11, 12, 47]. A more recent study showed that opening of cardiomyocyte Ca^2+^-activated K^+^ channels of the BK type (CMBK) is a critical modulator in remodelling following myocardial infarction (MI) using CMBK-knockout (KO) mice [24]. The study showed that more severe myocardial damage observed in the CMBK-deficient hearts after ischaemia/reperfusion (I/R) is accompanied by a significantly increased production of reactive oxygen species (ROS). Furthermore, the study showed that pharmacological agents that elevate intracellular cGMP no longer exhibited cardioprotective effects in CMBK-KO mice. Collectively, the authors proposed the GC–cGMP–CMBK pathway as a novel therapeutic target for preventing post-MI cardiac remodelling.

Although the precise mechanistic details how the cGMP–PKG pathway signals and interacts with downstream effectors to protect the heart remain to be characterised, a large number of preclinical studies have revealed the cardioprotective potency of the cGMP–PKG pathway by employing pharmacological tools or by manipulating relevant genes. Based on our limited understanding, the current therapeutic strategy for targeting this pathway is either by increasing cGMP biosynthesis (i.e. sGC activators or stimulators) or reducing cGMP’s catabolism (i.e. PDE inhibitors).

## Nitric oxide

Nitric oxide (NO) is a key upstream molecule able to increase intracellular cGMP. NO is generated as a by-product of the enzymatic conversion of l-arginine to l-citrulline by nitric oxide synthases (NOS) [60]. These include endothelial NOS (eNOS), neuronal NOS (nNOS) and inducible NOS (iNOS). All three isoforms are expressed in the cardiovascular system but have distinct subcellular localizations. eNOS and nNOS are constitutively expressed and work in a Ca^2+^-calmodulin-dependent manner, whereas iNOS is only expressed in response to inflammatory stimuli [7]. Although the cell-specific function of each isoform has not yet been fully elucidated, deletion of all three isoforms in mice resulted in the severe pathological phenotypes such as MI, spontaneous coronary artery disease and sudden cardiac death, demonstrating the cardioprotective importance of NOS in the cardiovascular system [45]. While l-arginine is the major substrate for endogenous NO production, nitrite, which can be elevated in the circulation by dietary intake, is another important source of NO [17, 51]. Nitrite can be converted non-enzymatically to NO via protonation at the low pH of the stomach [3]. Nitrite absorbed in blood and other tissues can be reduced to NO by xanthine oxidoreductase (XOR) or by a nitrite reductase activity of deoxygenated heme proteins such as deoxyhemoglobin. It is interesting to note that the rate of NO generation from nitrite is linearly dependent on reductions in oxygen and pH levels. Indeed exogenous nitrite reduced cardiac infarct size in mice subjected to myocardial I/R injury by upregulating NO [17, 29]. Due to nitrite’s high stability in the circulation, it is an important endocrine reservoir of NO. The beneficial effect of NO, at least in rodent models, has been widely reported, but there have also been discrepant results in some models. Several studies reported that NOS inhibition protects the hearts from I/R injury [21, 58, 66] and in one report exogenous NO administration actually worsened the functional recovery following I/R [57]. Furthermore, it has been shown that the infarct modulating effect of nitrite against myocardial I/R injury was very dose-dependent in mice [17]. Collectively, those studies suggest that the therapeutic effect of NO during I/R depends on dose, source, schedule and species.

Organic nitrates have been used as short-term treatment for acute MI, decompensated heart failure, and hypertensive crisis but their value in chronic treatment has been limited due to unfavourable hemodynamic effects, short plasma half-life, and the development of clinical tolerance. Much research has been devoted to overcoming these drawbacks of organic nitrates by developing a novel class of NO-releasing drugs. NO-releasing Aspirin (NCX4016), has been developed with a timed NO-releasing property [33] and has been extensively studied in various animal models. In addition to aspirin-related inhibition of platelet aggregation, chronic treatment with NCX4016 exerted an infarct-limiting effect in rabbits, pigs, and both normal and diabetic rats during myocardial I/R while native aspirin failed to protect against infarction [5, 53, 64]. Another class of novel NO-releasing drugs is the NO-statins such as NCX 6550 which showed anti-inflammatory, anti-proliferative and antiplatelet effects beyond the actions of statins alone [16, 48].

**Fig. 1:**
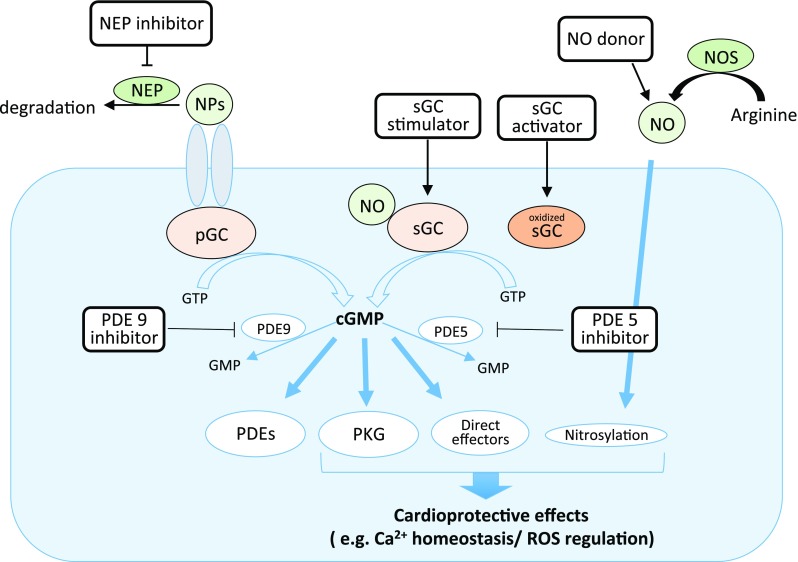
Hypothetical nitric oxide (NO)–protein kinase G (PKG) pathway. Abbreviations see text

The cardioprotective properties of NO are not limited to the cGMP–PKG-dependent pathway. It has been shown that NO can directly modify proteins through protein *S*-nitrosylation (SNO), which has recently emerged as an important post-translational protein modification and may offer great therapeutic potential in cardiovascular diseases [9, 10, 61]. Interestingly, mitochondria-selective *S*-nitrosylation by mitochondrial-targeted *S*-nitosothiol (MitoSNO) showed an infarct-reducing effect and improved cardiac function with myocardial I/R injury when it was administered 5 min before the onset of reperfusion. Complex I generates an excessive amount of ROS early in reperfusion due to succinate-driven reverse electron transport (RET) in mitochondria during I/R [49]. A cysteine residue (Cys39) in complex I becomes susceptible to *S*-nitrosylation in the ischemic heart muscle whenever the absence of respiration leads to low complex I activity. *S*-nitrosylation of the Cys39 in the ischemic cells by MitoSNO attenuated the reactivation of Complex I with reperfusion and the resulting lethal burst of ROS [10, 43]. The cardioprotective action of MitoSNO persisted in cardiomyocyte-specific PKG-I KO mice, indicating its independence of the cGMP–PKG pathway [10] (Fig. 1).

## sGC activators or stimulators

One major strategy to increase the intracellular pool of cGMP is to activate sGC [18]. NO can induce sGC’s activity upon its binding to the reduced Fe^2+^ heme moiety on its β-subunit. However, oxidation of the heme moiety under pathological conditions during oxidative stress, such as diabetes, can result in reduced sensitivity of sGC to NO. Also the reduced bioavailability of NO associated with endothelial dysfunction is another factor that can limit the activity of sGC. Two classes of small molecule compounds have been developed to directly target sGC. sGC stimulators increase the catalytic activity of sGC with a reduced Fe^2+^ heme moiety. These compounds work synergistically with NO. On the other hand, sGC activators can activate the enzyme when the heme moiety is oxidised or missing. sGC activators can also trigger cGMP synthesis independently of NO [18]. Both classes of drugs, sGC activators (e.g. BAY58-2667—Cinaciguat and HMR-1766—Ataciguat) [23, 36, 56] and stimulators (e.g. BAY63-6521—Riociguat, BAY60-4552, and BAY1021189—Vericiguat) [2, 42], have shown promising results in preclinical and clinical studies. Among them, Vericiguat has a dual mode of action. It sensitises sGC to endogenous NO by promoting NO–sGC binding and it also activates sGC independently of NO. Vericiguat has been structurally optimised for chronic use in heart failure (HF) patients, allowing once-a-day dosage with low pharmacokinetic variability [22]. In a Phase IIb dose-finding study, the SOCRATES-REDUCED trial, an exploratory analysis showed that Vericiguat improved left ventricular ejection fraction (EF) and reduced NT-proBNP (biomarker for heart failure) at the highest dose in subjects with reduced ejection fraction heart failure (HFrEF) [25]. Vericiguat entered into a phase III clinical trial, the VICTORIA trial, in 2016 in HFrEF patients [1].

## Natriuretic peptides

Natriuretic peptides (NPs) augment the intracellular level of cGMP via pGC [46]. NPs such as ANP and BNP are upregulated to compensate loss of function in failing hearts. Their elevated plasma levels also serve as well-established biomarkers for heart failure. NPs have shown multiple pharmacological effects including diuresis, natriuresis, vasodilation and inhibition of the renin-angiotensin and aldosterone systems. There are a number of preclinical studies showing that elevating NPs results in cardioprotection against I/R injury and in hypertrophy models [8, 13, 46, 55]. Clinically, less data are available, however, the J-WIND-ANP trial showed promising results [35]. A continuous infusion of ANP for 3 days following reperfusion led to a 14.7% reduction in infarct size with reduced total creatine kinase (CK) release (66,459.9 IU/ml per h in the treated group vs. 77,878.9 IU/ml per h in controls) and a small but significant improvement in EF compared to the control group. In 2014, the PARADIGM-HF trial has shown that administration of Sacubitril, a first-in-class neprilysin inhibitor that interferes with NP degradation, resulted in a significant improvement in patients with HFrEF when it was given together with the angiotensin II receptor blocker, Valsartan [32, 41]. Following the successful clinical trials, Sacubitril/Valsartan (Entresto™) was approved for the treatment of HFrEF.

## PDE inhibitors

The intracellular pool of cGMP is tightly regulated by PDEs, enzymes degrading cGMP, as an important part of cGMP–PKG pathway. There are 6 different PDE isoforms, PDE 1, 2, 3, 4, 5 and 9, that are expressed in the heart and are responsible for cGMP catabolism in the cardiovascular system. It is reported that PDE5 is predominantly responsible for hydrolysis of cGMP produced by sGC [63, 65]. PKG-I phosphorylates and activates PDE-5 by increasing its affinity to cGMP, thereby enhancing cGMP hydrolysis. This cGMP–PKG–PDE-5 signalling works efficiently as a negative feedback regulation maintaining the physiological cGMP homeostasis. The expression of PDE 5 is very low and mainly confined to smooth muscle cells under physiological conditions but it was found to be upregulated in ischemic and failing myocardium [44, 50, 59]. With such pathological changes, use of a PDE-5 inhibitor such as sildenafil, which is widely prescribed for the treatment of erectile dysfunction, has shown promising results against I/R injury, cardiac hypertrophy and heart failure in both preclinical and clinical settings [14, 20, 26, 38, 62]. A recent study has also demonstrated that co-treatment of a PDE-5 inhibitor, tadalafil, has a synergistic effect with the protection afforded by inhaled NO starting 30 min before reperfusion and continued for 20 min during reperfusion. This additional protection against infarction was accompanied with a significant increase of cardiac cGMP levels [40]. Although the mechanism of its protection is not fully elucidated yet, multiple studies have demonstrated that the cardioprotective effect of PDE-5 inhibitors is PKG pathway-dependent using pharmacological PKG inhibitors or selectively knocking down PKG in cardiomyocytes [15, 54]. A number of small scale clinical studies have shown favourable effects of PDE-5 inhibitors such as improved hemodynamics, left ventricular (LV) diastolic function and right ventricular (RV) systolic function in patients with heart failure with preserved ejection fraction (HFpEF). However, a recent multicentre, double-blinded, randomised, controlled trial, RELAX, failed to confirm a beneficial effect of sildenafil against HFpEF [52]. Based on evidence from prior clinical studies and the limitations of the RELAX trial, the authors suggested that only HF patients with reactive pulmonary hypertension are likely to benefit [34].

Apart from PDE-5, Lee et al. recently suggested PDE9A as a novel therapeutic target against heart failure [37]. PDE9A is expressed in cardiomyocytes and further upregulated by hypertrophy and heart failure. Interestingly, the study showed that PDE9A regulates cGMP produced by the NP–pGC pathway rather than the NO–sGC pathway. Genetic or pharmacological inhibition of PDE9A activity improved cardiac function in mice subjected to pressure overload hypertrophy by severe transverse aortic constriction (TAC). The authors proposed that PDE9A as an alternative therapeutic approach which might be effective alone or in combination with other drugs for treatment of heart failure.

## Summary

Despite promising results from many preclinical studies suggesting a cardioprotective effect of cGMP–PKG signalling, a number of clinical studies evaluating GC modulators and PDE 5 inhibitors have failed to show the efficacy in large cohorts. The current difficulty in translating preclinical observation to clinical efficacy might be due to the distinct disease states derived from differences between preclinical animal models and humans [19]. For instance, subjects enrolled in clinical studies evaluating potential therapeutic tools against heart failure are likely complicated with other age-related diseases such as metabolic syndrome and other cardiovascular problems, which are often absent in preclinical animal models. Under such complex pathological condition, the efficacy of GC modulators or PDE-5 inhibitor could be limited due to the reduced sensitivity of GC to NO or the limited availability of NO, as discussed above. Based on our current understanding the complexity of such age-related diseases, employing combination therapy strategy targeting multiple mechanisms involved in the cGMP–PKG pathway, for example, targeting both NO-independent NP–pGC–cGMP and NO-dependent sCG–cGMP pathways, might be able to shed some light in the quest for new therapeutic tools against heart failure.
